# Assessing feasibility of a modified same-day test-and-treat model for hepatitis C among rural people who inject drugs

**DOI:** 10.1186/s12954-023-00780-3

**Published:** 2023-04-12

**Authors:** Muhammad Radzi Abu Hassan, Huan-Keat Chan, Mahani Nordin, Ranimah Yahya, Wan Ruzilasalwa Wan Sulaiman, Siti Aminah Akbar Merican, Darisah Lah, Xiaohui Sem, Sonjelle Shilton

**Affiliations:** 1 Clinical Research Center, Sultanah Bahiyah Hospital, 05460 Alor Setar, Kedah Malaysia; 2 Public Health Division, State Health Department, Kuala Terengganu, Terengganu Malaysia; 3 Rahmat Health Clinic, Setiu, Terengganu Malaysia; 4 Kuala Dungun Health Clinic, Dungun, Terengganu Malaysia; 5 Kuala Berang Health Clinic, Hulu Terengganu, Terengganu Malaysia; 6 Bukit Tunggal Health Clinic, Kuala Nerus, Terengganu Malaysia; 7 FIND, Geneva, Switzerland

**Keywords:** Antiviral agents, hepatitis C, Malaysia, Public health, Health services accessibility

## Abstract

**Background:**

Despite advancements in hepatitis C virus (HCV) treatment, low uptake among hard-to-reach populations remains a global issue. The current study aimed to assess the feasibility of a modified same-day test-and-treat model in improving HCV care for people who inject drugs (PWID) living in resource-constrained rural areas.

**Methods:**

A pilot study was conducted in four primary healthcare (PHC) centers in Malaysia. The model's key features included on-site HCV ribonucleic acid (RNA) testing using a shared GeneXpert® system; noninvasive biomarkers for cirrhosis diagnosis; and extended care to PWID referred from nearby PHC centers and outreach programs. The feasibility assessment focused on three aspects of the model: demand (i.e., uptake of HCV RNA testing and treatment), implementation (i.e., achievement of each step in the HCV care cascade), and practicality (i.e., ability to identify PWID with HCV and expedite treatment initiation despite resource constraints).

**Results:**

A total of 199 anti-HCV-positive PWID were recruited. They demonstrated high demand for HCV care, with a 100% uptake of HCV RNA testing and 97.4% uptake of direct-acting antiviral treatment. The rates of HCV RNA positivity (78.4%) and sustained virologic response (92.2%) were comparable to standard practice, indicating the successful implementation of the model. The model was also practical, as it covered non-opioid-substitution-therapy-receiving individuals and enabled same-day treatment in 71.1% of the participants.

**Conclusions:**

The modified same-day test-and-treat model is feasible in improving HCV care for rural PWID. The study finding suggests its potential for wider adoption in HCV care for hard-to-reach populations.

## Background

Hepatitis C virus (HCV) infection continues to pose a significant threat to global health, affecting 58 million people and causing 290,000 deaths every year [1]. The World Health Organization (WHO) has set a target to diagnose 90% of individuals living with HCV and treat 80% of them by 2030 [2]. To achieve this goal, WHO has called for simplification of HCV care and increased access to affordable treatment [3]. However, many countries face challenges in treating vulnerable and hard-to-reach populations [4]. Additionally, the financial burden of diagnostics and medication remains a major barrier to treatment scale-up efforts, particularly in low- and middle-income countries (LMICs) [5, 6].

The introduction of direct-acting antivirals (DAAs) has transformed the management of HCV over the past decade [7]. According to a systematic review, a few pan-genotypic DAA combinations, including sofosbuvir/daclatasvir, have demonstrated sustained virologic response rates of over 94% [8]. DAAs are generally safe, even for individuals with human immunodeficiency virus (HIV)/HCV co-infection or cirrhosis [9, 10]. The availability of DAAs and rapid anti-HCV test kits has also enabled the initiation of treatment at the primary care level, particularly for non-cirrhotic HCV cases [7–11].

Malaysia bears a moderate burden of HCV, with approximately 1.9% to 2.5% of its population living with the disease [12, 13]. As in several LMICs, the high cost of DAAs had been a significant impediment to scaling up DAA-based treatment in Malaysia [14]. To overcome the limitations of accessing patented DAAs, Malaysia took the pioneering step of issuing a compulsory license to authorize the import and use of generic sofosbuvir in 2017 [14, 15]. Sofosbuvir/daclatasvir was subsequently selected to be the standard treatment regimen for HCV in public hospitals, resulting in an SVR rate of 95.4% [16]. By implementing compulsory licensing on sofosbuvir, Malaysia successfully made free HCV treatment available through the public health system without incurring a substantial budgetary strain [17, 18].

In 2019, the decentralization of HCV care in Malaysia allowed for access to rapid anti-HCV testing and DAA-based treatment for key populations at risk of HCV in primary healthcare (PHC) centers operated by the Ministry of Health [19]. While hospitals continue to serve as referral centers for those with cirrhosis and offer laboratory support for HCV ribonucleic acid (RNA) testing, not all people who inject drugs (PWID) seek medical care despite the high incidence of HCV [20, 21]. Currently, active case-finding for HCV in Malaysia focuses on individuals receiving opioid substitution therapy (OST) in PHC centers [21, 22]. Furthermore, logistical barriers associated with specimen delivery and lengthy laboratory turnaround times in PHC centers, especially in remote locations, remain unaddressed [21].

**Fig. 1:**
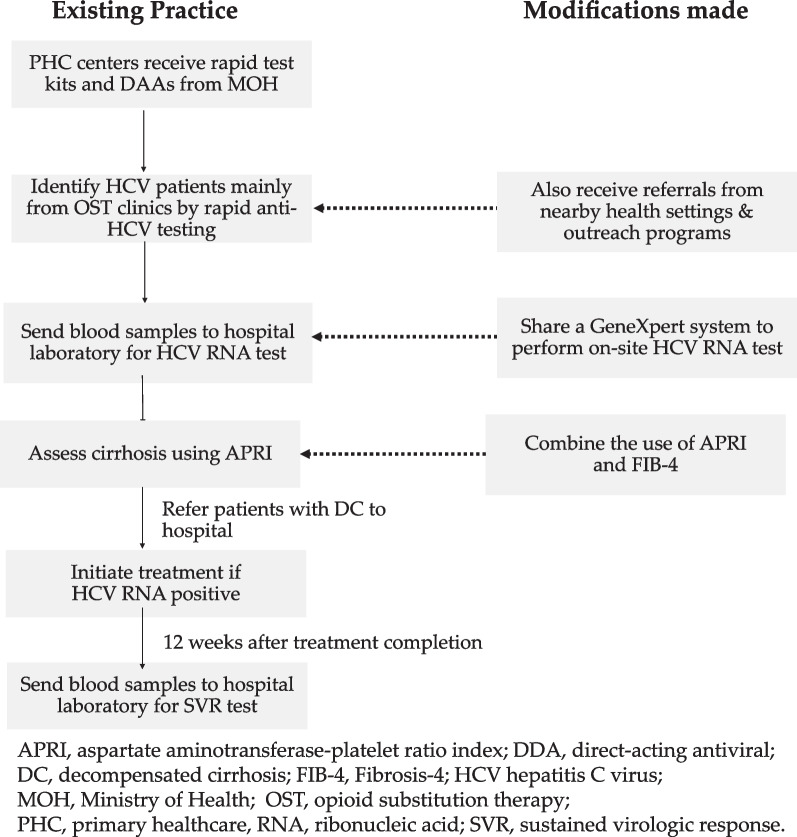
Modifications made to the existing hepatitis C care practice

While the evidence supporting the effectiveness of decentralized HCV care is established, there is still limited information on its feasibility in LMICs, particularly for hard-to-reach populations such as PWID. Less than one-fifth of the studies on decentralized HCV care focused on LMICs [10]. To address these limitations, a modified same-day test-and-treat model for HCV was developed in Malaysia (see Fig. 1). The Ministry of Health collaborated with FIND, a non-profit organization dedicated to ensuring equitable access to reliable diagnostics, to create this model. The current study aimed to assess the feasibility of the model in improving HCV care for PWID in resource-constrained rural areas. Additionally, it sought to investigate any differences in achieving each step of the HCV care cascade between existing patients of PHC centers and those referred from outside the centers.

## Methods

### Study settings

Terengganu, located on the east coast of Peninsular Malaysia, has a population of 1.04 million, with almost half living in rural areas [23]. The modified same-day test-and-treat model was piloted in four rural areas of Terengganu (i.e., Setiu, Kuala Berang, Kuala Dungun and Bukit Tunggal). One PHC center was selected from each area as the study site. Each study site was staffed by a medical team led by a family physician experienced in providing HCV care. The team was responsible for implementing the modified same-day test-and-treat model.

### Modified same-day test-and-treat model

The modified same-day test-and-treat model was adapted from the existing practice in Malaysia (see Fig. 1) [19]. The implementation of this model in the current study was led by the Ministry of Health and did not receive any external funding. The model incorporated resource and network sharing. Instead of using hospital laboratories, the four study sites shared a GeneXpert® system (Cepheid) equipped with four modules to perform WHO-prequalified, on-site HCV RNA amplification testing [24]. The GeneXpert® system was rotated among the study sites every two to four months, and recruitment was limited to the period when the GeneXpert® system was available at a study site. The duration of placement was based on the estimated time required to treat as many PWID within a 5 km radius of each study site as determined by family physicians. The estimates factored in the additional time required for case finding, which was influenced by the effects of the COVID-19 pandemic. In addition to active case-finding in OST clinics, nearby PHC centers and community-based organizations (CBOs) were encouraged to refer potential participants to the study sites. Communication between organizations was coordinated by the state public health officer.

### Study population

Only individuals who met the following criteria were eligible for the current study: (i) were at least 18 years of age, (ii) had a history of intravenous drug use, (iii) tested positive for anti-HCV, and (iv) were naïve to DAA-based treatment. The required sample size for the study was 194, as estimated based on a pilot project of HCV care decentralization in Malaysia, which reported that 83.8% of anti-HCV-positive participants were HCV RNA positive [19]. The formula used for the sample size calculation was the single proportion estimation formula [25], with a fixed level of confidence of 90% and a precision of 5%.

### Study procedures

The current study followed a longitudinal and observational design. All medical procedures were performed by the staff of the study sites. Data collection was conducted by a trained research assistant. Recruitment took place between 1 February and 31 December 2021. The participants were first briefed about the study and screened for eligibility. Demographic and medical history data were collected. If no record of HCV screening was available, their anti-HCV status was assessed using a rapid test kit approved by the Medical Device Authority in Malaysia [26]. Their intravenous drug use history was determined based on previous urine test results or self-reporting.

Each participant provided a venous blood sample (10 mL) for HCV RNA testing, complete blood count and liver function testing. Laboratory technicians in the study sites performed all tests immediately after blood sample collection, and the results were used to guide family physicians in treatment decisions. Participants whose HCV RNA test results were invalid more than once were referred to a family physician for standard care and were excluded from further analysis. Additional blood samples were also collected from participants with unclear HIV and hepatitis B virus (HBV) status for rapid and confirmatory (laboratory-based enzyme immunoassay) testing.

The cirrhosis status of participants was assessed using the combination of aspartate aminotransferase-platelet ratio index (APRI) and Fibrosis-4 (FIB-4) score [27]. An APRI ≥ 1.5 indicated cirrhosis. The FIB-4 score (> 3.25) was only used to determine the presence of cirrhosis if the APRI fell between 1.0 and 1.5. The combined use of the two biomarkers in such a sequential manner was shown to produce higher sensitivity and specificity for the diagnosis of cirrhosis associated with HCV [28].

Treatment initiation was recommended only if the HCV RNA test was positive. The recommended treatment duration was 12 weeks for participants with no cirrhosis and 24 weeks for those with compensated cirrhosis [7, 27]. Participants with HIV/HCV co-infection only received DAAs if they were receiving antiretrovirals [27]. Participants with HBV/HCV co-infection or symptoms of decompensated cirrhosis were referred to the state hospital for specialized care. It was aimed that all test results would be made available within 3 h of recruitment, and treatment was initiated on the same day as the HCV RNA testing. However, an additional visit was required if additional investigations were necessary or if participants refused to wait. The subsequent visit occurred 12 weeks after the last dose of DAAs. Treatment completion was confirmed by self-reporting and prescription-refill records. Venous blood samples were collected and delivered to the state hospital for SVR testing. Participants who failed to achieve an SVR were contacted by phone and referred to the state hospital for further management.

### Outcomes

The feasibility assessment focused on three aspects of the model: demand (i.e., uptake of HCV RNA testing and treatment), implementation (i.e., achievement of each step in the HCV care cascade), and practicality (i.e., ability to identify PWID with HCV and expedite treatment initiation despite resource constraints) [29]. The study outcomes included the following proportions of participants: (i) those who underwent on-site HCV RNA testing; (ii) those with a valid HCV RNA test result; (iii) those who tested positive for HCV RNA; (iv) those who received DAA-based treatment; (v) those who started treatment on the same day as testing; (vi) those who completed treatment; (vii) those who had SVR testing performed 12 weeks after treatment completion; and (viii) those who achieved SVR.

To determine the feasibility of the model, the study team assessed whether its outcomes were comparable to those achieved in standard HCV care in Malaysia. Based on a review of relevant studies [16, 19] and local data obtained from the Ministry of Health, the team considered the model feasible if it met the following criteria: (i) at least 70% of PWID who tested positive for anti-HCV received HCV RNA testing; (ii) only a small fraction (< 5%) of PWID repeated HCV RNA testing due to an initial invalid result; (iii) at least 70% of PWID who underwent HCV RNA testing had a detectable level of HCV RNA; (iv) at least 60% of eligible PWID received treatment; (v) at least 60% of PWID who were treated started treatment on the same day; (vi) at least 70% of PWID who started treatment completed it; (vii) at least 60% of PWID who completed treatment underwent SVR testing; (viii) at least 80% of PWID achieved SVR after completing treatment; and (ix) at least 30% of the PWID who received treatment under the model were referred from sources other than the study sites.

In addition, the duration between HCV RNA testing and treatment initiation was measured, and demographics and achievement in each step of the HCV care cascade were compared between participants identified from the four study sites and those referred from other sources. Although this study was not specifically designed to undertake a thorough cost analysis of the model, the financial implications of its implementation were estimated from the provider’s perspective by computing the costs incurred by activities ranging from screening, confirmatory testing, cirrhosis assessment, treatment to SVR testing. It was assumed that the utilization of existing resources including the facility and personnel did not result in additional expenses.

### Statistical analysis

Descriptive statistics were used to summarize participant characteristics and results of the test-and-treat model implementation. Categorical variables were presented as frequencies and percentages, while numerical variables were expressed as means and standard deviations or medians and interquartile ranges. The uptake of on-site HCV RNA testing was analyzed for the entire study population. Analysis of HCV RNA status was conducted in participants who underwent testing, while treatment uptake was analyzed in those who were HCV RNA positive. Treatment initiation and completion status were analyzed in participants who received treatment, and SVR assessment was restricted to those who completed treatment and underwent testing. The Pearson’s Chi-square, Fisher’s exact or independent t-test was used to compare differences between participants identified from the four study sites and those referred from other sources. All data were analyzed using SPSS® version 26 (IBM, New York), and a p value < 0.05 was considered significant.

## Results

### Participant characteristics

**Fig. 2:**
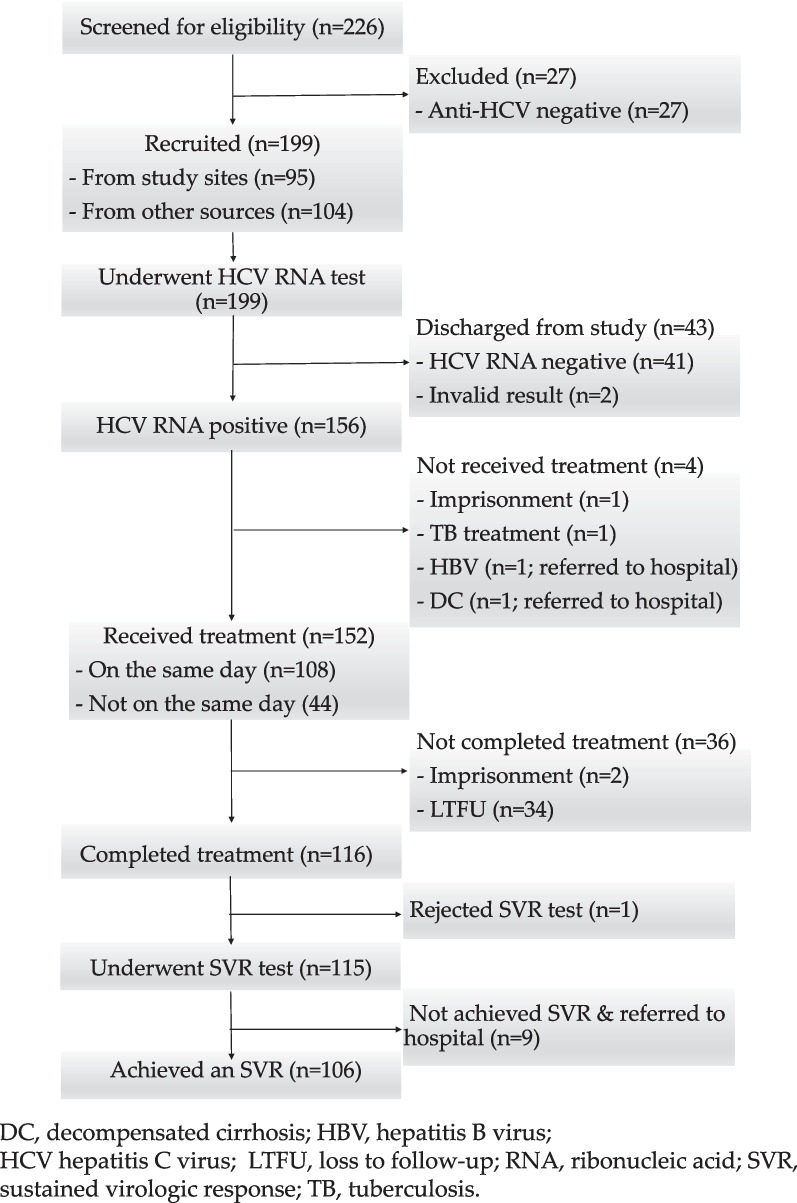
Progression of participants along the hepatitis C care cascade

A total of 226 individuals were screened for eligibility, and 27 were excluded for testing negative for anti-HCV (see Fig. 2). The number of participants recruited varied across the study sites, ranging from 22 to 79 (see Table 1). All study participants were male and of Malay ethnicity, with a mean age of 44 years (SD = 6.5). Approximately one-fifth of participants were living with HIV, and only 1.0% were living with HBV. HIV/HCV co-infection was more common among participants identified from the study sites compared to those referred from other sources (27.4% versus 11.5%; p = 0.005). All participants living with HIV were receiving antiretrovirals. Over 40% of participants had their anti-HCV status tested at least one year prior to the study.

**Table 1:** Participant characteristics

Characteristics	Overall	Participants identified from study sites	Participants referred from other sources	*p*
Study site locations, n (%)				
Rahmat	79/199 (39.7)	36/95 (37.9)	43/104 (41.3)	< 0.001^a^
Kuala Dungun	22/199 (11.1)	22/95 (23.2)	0/104 (0)	
Bukit Tunggal	47/199 (23.6)	19/95 (20.0)	28/104 (26.9)	
Kuala Berang	51/199 (25.6)	18/95 (18.9)	33/104 (31.7)	
Age, years, mean (SD)	44.0 (6.5)	43.9 (6.5)	44.0 (6.6)	0.894^b^
Gender, n (%)				
Male	199/199 (100)	95/95 (100)	104/104 (100)	–
Ethnicity, n (%)				
Malay	199/199 (100)	95/95 (100)	104/104 (100)	–
Living with HIV, n (%)	38/199 (19.1)	26/95 (27.4)	12/104 (11.5)	0.005^a^
Living with HBV, n (%)	2/199 (1.0)	0/95 (0)	2/104 (1.9)	0.499^c^
Timepoint of anti-HCV test, n (%)				
Within 1 year	119/199 (59.8)	55/95 (57.9)	64/104 (61.5)	0.601^a^
≥ 1 year	80/199 (40.2)	40/95 (42.1)	40/104 (38.5)	

*HBV* hepatitis B virus, *HIV* human immunodeficiency virus, *n* size of sample, *SD* standard deviation

^a^Pearson’s Chi-square test

^b^Independent t-test

^b^Fisher’s exact test

### Results of test-and-treat model implementation

**Fig. 3:**
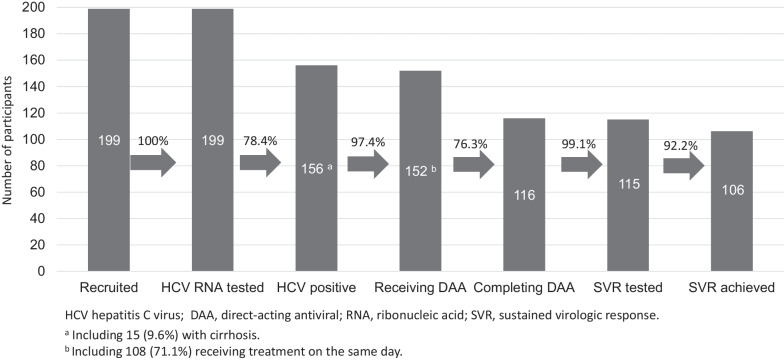
Achievement of each step in the hepatitis C care cascade

The model met all the evaluation criteria, indicating its feasibility. Slightly less than half (48.7%) of the participants were recruited from the study sites, while the remaining participants were referred from other sources. All 199 study participants underwent on-site HCV RNA testing (see Fig. 3), and 78.4% (156/199) of them had a positive result. Among the participants who were HCV RNA positive, only 9.6% (15/156) had cirrhosis, and 97.4% (152/156) received treatment. Two participants did not initiate treatment due to imprisonment and ongoing antituberculosis therapy, while two others were referred to the state hospital for specialized care. Of the participants who received treatment, 71.1% (108/152) had treatment initiated on the same day as the HCV RNA testing, and 76.3% (116/152) completed treatment. Participants referred from other sources were more likely to have same-day treatment initiation (80.2% versus 60.6%; p = 0.008), while those identified from the study sites were more likely to complete treatment (85.9% versus 67.9%; p = 0.009) (see Table 2). The median duration between SVR RNA testing and treatment initiation was 0 days. As of September 30, 2022, 99.1% (115/116) of the participants who completed treatment had an SVR test result available, with only one participant refusing reevaluation. The SVR rate was 92.2% (106/115). The estimated cost of testing and treating an HCV-infected individual without cirrhosis under the model was USD356.54, representing an increase of approximately 5.8% compared to the cost under standard care (USD337.04 per patient).

**Table 2:** Results of the test-and-treat model implementation by participant source

Outcomes	Participants identified from study sites	Participants referred from other sources	*p*
Underwent an on-site viral load test, n (%)	95/95 (100)	104/104 (100)	–
Viral load test result, n (%)			
Positive	71/95 (74.7)	85/104 (81.7)	0.536^a^
Negative	23/95 (24.2)	18/104 (17.3)	
Invalid	1/95 (1.1)	1/104 (1.0)	
Had cirrhosis, n (%)	8/71 (11.3)	7/85 (8.2)^b^	0.522^c^
Received DAA-based treatment, n (%)	71/71 (100)	81/85 (95.3)^d^	0.126^a^
Referred to the state hospital for specialized care, n (%)	0/71 (100)	2/85 (2.4)^e^	0.501^a^
Received DAA-based treatment on the same day as the viral load test, n (%)	43/71 (60.6)	65/81 (80.2)	0.008^c^
Completed treatment, n (%)	61/71 (85.9)	55/81 (67.9)	0.009^c^
Reasons of incomplete treatment, n (%)			
Imprisonment	1/10 (10.0)	1/26 (3.6)	0.484^a^
LTFU	9/10 (90.0)	25/26 (96.2)	
Had an SVR test result, n (%)	61/61 (100)	54/55 (98.2)	0.474^a^
Achieved SVR, n (%)	56/61 (91.8)	50/54 (92.6)	> 0.95^a^

*DAA* direct-acting antiviral, *n* size of sample, *LTFU* loss to follow-up, *SD* standard deviation, *SVR* sustained virologic response

^a^Fisher’s exact test

^b^One study participant showed symptoms of decompensated cirrhosis

^c^Pearson’s Chi-square test

^d^Four participants did not receive DAA-based treatment due to imprisonment (n = 1), receiving antituberculotic treatment at the same time (n = 1), hepatitis B co-infection (n = 1) and showing symptoms of decompensated cirrhosis (n = 1)

^e^Due to hepatitis B co-infection (n = 1) and showing symptoms of decompensated cirrhosis (n = 1)

## Discussion

The current study found that the modified same-day HCV test-and-treat model was feasible and effective in improving HCV care among PWID from resource-constrained rural areas. The strength of the current study lies in the successful integration of the model with the existing public health system. While similar test-and-treat models in Myanmar and Egypt were implemented through externally funded access programs [30, 31], the current study suggests that a government-led initiative could also be effective in scaling up treatment for people living with HCV from LMICs. Such models are also likely to be more sustainable with political buy-in and adequate support for the supply of diagnostics and medicines from the government [32].

A high demand for both HCV confirmatory testing and treatment among participants was observed. Similar to two studies with similar test-and-treat models [30, 31], all participants in the current study underwent HCV RNA testing, and all HCV-RNA positive participants except for a few with complicated conditions received DAA-based treatment. The results imply that DAAs are widely accepted among PWID, possibly due to their established efficacy and safety profiles [33]. It is noteworthy that more than 40% of participants had their anti-HCV status tested at least one year prior to the study, indicating the failure to promptly confirm HCV diagnosis in a significant proportion of anti-HCV-positive PWID after the rapid screening test in Malaysia. In fact, logistical challenges, such as specimen delivery to hospitals and lengthy laboratory turnaround times, have historically hindered the timely provision of HCV care [21]. Nevertheless, the use of the GeneXpert® system to perform on-site HCV RNA testing in PHC centers in the current study overcame these barriers.

The modified same-day test-and-treat model was also implemented successfully, as demonstrated by the strong performance achieved in almost all steps of the HCV care cascade. There were only two incidents of repeated invalid tests for HCV RNA, which were likely due to a failure in the internal quality control of the GeneXpert® system [24]. The fact that both the individuals involved still received treatment indicated that the standard HCV care, which was characterized by laboratory-based confirmatory testing, complemented the new same-day test-and-treat model well. The new model was also as effective as the standard care in identifying PWID who were HCV-RNA-positive [19]. Family physicians in Malaysia were found to be capable of managing PWID with HCV effectively, including cases complicated by cirrhosis and HIV infection, and initiating DAA-based treatment when necessary, similar to the experience in Cambodia [30]. Furthermore, the participants achieved an SVR rate that was almost as high as that reported by hospitals when sofosbuvir-based treatment was first made available in Malaysia [16]. These positive findings throughout the care cascade increase in the confidence of the Ministry of Health in extending HCV care to more hard-to-reach populations in the country.

Although HCV treatment remains costly in many LMICs [34], the current study has demonstrated how a practical test-and-treat model could improve HCV care in remote areas with resource constraints. While the GeneXpert® system has mainly been adopted with the help of funding from international non-profit organizations [30, 35], its use for HCV care in LMICs faces challenges due to high acquisition and maintenance costs [36]. However, the current study suggests that sharing a GeneXpert® system among PHC centers is a viable option to reduce financial burden without compromising HCV care. In Malaysia, HCV care activities are centrally coordinated by a public health officer, and at least one PHC center in each state is equipped with a GeneXpert® system. Hence, rotating the GeneXpert® system among various PHC centers, which would require this approach to treat hard-to-reach HCV populations, could be a highly feasible long-term solution, provided proper planning and leadership are in place.

More than 70% of HCV-RNA-positive participants were found to initiate treatment on the same day as the HCV RNA testing. While the finding is encouraging, it is important to note that the remaining eligible participants did not receive immediate treatment after testing. Although the reasons were not investigated, it was observed that a significant proportion of participants recruited from the study sites was not willing to wait for treatment on the same day. This was particularly true for those under OST, who decided to initiate treatment later when returning to PHC centers. Therefore, while aiming to deliver same-day treatment, it is crucial to incorporate some degree of flexibility for HCV patients in the future. Additionally, the current study showed that HCV care for hard-to-reach populations could be improved through partnerships between the Ministry of Health and CBOs. This approach was also highly recommended in a qualitative study involving key stakeholders of HCV care in Malaysia [21]. The modified same-day test-and-treat model used in the current study successfully expanded the case-finding to cover not only PHC centers but also nearby health settings and CBO-led outreach programs.

Nonetheless, in contrast to other similar studies [30, 31, 35], the current study reported only a moderate treatment completion rate, which can be expected as it was conducted in real-world public PHC centers without the aid of external workforce and resources. The primary reason for incomplete treatment in the current study was loss to follow-up, with participants referred from other sources being more likely not to complete their treatment than those identified from the study sites. The finding is understandable, as the distance between healthcare facilities and residences may contribute to loss to follow-up among people living with HCV [37]. Apart from attempting to locate and retrieve those who were lost to follow-up, it is crucial for physicians to address the social and psychological factors that may influence their attitudes toward treatment in the future [38].

It was also discovered that the new model resulted in a slight increase in the cost of testing and treating an individual living with HCV by approximately 5.8%. The primary factor contributing to this increase was the higher cost of on-site HCV RNA testing (USD35.5 per test) compared to HCV RNA testing in hospital laboratories (USD16 per test). However, it is possible that the actual cost difference is smaller, as the estimation did not consider logistical factors such as preparation of specimens and transportation involved in delivering them to hospital laboratories under standard care. Although annual allocation for HCV by the Ministry of Health may be sufficient to absorb the increased cost in Malaysia, the long-term financial impact needs to be evaluated, particularly with the expected increase in the number of people living with HCV to be diagnosed and treated. It is also essential to factor in expenses on the maintenance of the GeneXpert® system and training, which were covered by the distributor in this study, into future cost estimates. An alternative approach would be to reserve the new model for PHC centers which struggle with retaining people living with HCV for care, rather than completely replacing standard care.

There were some limitations in the current study. The modified same-day test-and-treat model was only implemented in one state, and the participants were mainly males and Malays, as these were the predominant groups of PWID in the state. While the four selected study sites had access to stable electricity for the GeneXpert® system operation and were not affected by any disruptive events, such as floods, during the study period, it is uncertain whether the same model would be feasible in PHC centers with less favorable conditions. Although the state public health officer, family physicians and members of CBOs demonstrated a strong commitment to the study, it also remains unclear whether the findings can be generalized to other regions and populations. Additionally, the duration of GeneXpert® system placement in each study site was solely determined by the estimates of family physicians. However, PWID who were not treated during the study period could still receive standard HCV care at PHC centers.

## Conclusions

The findings of the current study highlight the feasibility of a modified same-day test-and-treat model in enhancing HCV care for PWID residing in rural areas. Further research should investigate the factors influencing its implementation across different geographical regions and optimize HCV testing strategies accordingly.

## Data Availability

The datasets generated and analyzed during the current study are not publicly available due to privacy concerns but are available from the corresponding author on reasonable request.

## Abbreviations

CBO: Community-based organization
DAA: Direct-acting antiviral
HBV: Hepatitis B virus
HCV: Hepatitis C virus
HIV: Human immunodeficiency virus
LMICs: Low- and middle-income countries
SVR: Sustained virologic response
PHC: Public healthcare
PWID: People who inject drugs
RNA: Ribonucleic acid
WHO: World Health Organization
