# Correction: Assessing feasibility of a modified same-day test-and-treat model for hepatitis C among rural people who inject drugs

**DOI:** 10.1186/s12954-023-00800-2

**Published:** 2023-05-19

**Authors:** Muhammad Radzi Abu Hassan, Huan-Keat Chan, Mahani Nordin, Ranimah Yahya, Wan Ruzilasalwa Wan Sulaiman, Siti Aminah Akbar Merican, Darisah Lah, Xiaohui Sem, Sonjelle Shilton

**Affiliations:** 1 Clinical Research Centre, Sultanah Bahiyah Hospital, Alor Setar, Kedah Malaysia; 2 Public Health Division, State Health Department, Kuala Terengganu, Terengganu Malaysia; 3 Rahmat Health Clinic, Chalok, Terengganu Malaysia; 4 Kuala Dungun Health Clinic, Kuala Dungun, Terengganu Malaysia; 5 Kuala Berang Health Clinic, Kuala Berang, Terengganu Malaysia; 6 Bukit Tunggal Health Clinic, Kuala Terengganu, Terengganu Malaysia; 7 FIND, Geneva, Switzerland

**Correction to: Harm Reduction Journal (2023) 20:48** **https://doi.org/10.1186/s12954-023-00780-3**

Following publication of the original article [1], in this article the affiliation details for Author A were incorrectly given as ‘Public Health Division, State Health Department, Alor Setar, Kedah, Malaysia’ but should have been ‘Public Health Division, State Health Department, Kuala Terengganu, Terengganu, Malaysia’.

The original article has been corrected.
